# Tet3 mediates stable glucocorticoid-induced alterations in DNA methylation and *Dnmt3a/Dkk1* expression in neural progenitors

**DOI:** 10.1038/cddis.2015.159

**Published:** 2015-06-18

**Authors:** R Bose, S Spulber, P Kilian, N Heldring, P Lönnerberg, A Johnsson, M Conti, O Hermanson, S Ceccatelli

**Affiliations:** 1 Department of Neuroscience, Karolinska Institutet, Stockholm, Sweden; 2 Department of Medical Biochemistry and Biophysics, Karolinska Institutet, Stockholm, Sweden

## Abstract

Developmental exposure to excess glucocorticoids (GCs) has harmful neurodevelopmental effects, which include persistent alterations in the differentiation potential of embryonic neural stem cells (NSCs). The mechanisms, however, are largely unknown. Here, we investigated the effects of dexamethasone (Dex, a synthetic GC analog) by MeDIP-like genome-wide analysis of differentially methylated DNA regions (DMRs) in NSCs isolated from embryonic rat cortices. We found that Dex-induced genome-wide DNA hypomethylation in the NSCs *in vitro*. Similarly, *in utero* exposure to Dex resulted in global DNA hypomethylation in the cerebral cortex of 3-day-old mouse pups. Dex-exposed NSCs displayed stable changes in the expression of the DNA methyltransferase Dnmt3a, and Dkk1, an essential factor for neuronal differentiation. These alterations were dependent on Tet3 upregulation. In conclusion, we propose that GCs elicit strong and persistent effects on DNA methylation in NSCs with Tet3 playing an essential role in the regulation of Dnmt3a and Dkk1. Noteworthy is the occurrence of similar changes in Dnmt3a and Dkk1 gene expression after exposure to excess GC *in vivo*.

Glucocorticoid (GC) hormones are critical for the terminal maturation of organs, but fetal exposure to high levels of GCs have detrimental effects on the development of the nervous system, including impaired neurogenesis, alterations of the hypothalamic-pituitary-adrenal axis, and behavioral changes.^1, 2, 3, 4, 5, 6, 7, 8^ The fetus is protected from surges of GC by placental enzymes (namely 11bHSD2) that convert circulating GC into inactive, water soluble metabolites.^9^ Conditions that are associated with high fetal GC levels include severe maternal stress, placental failure, and exogenous administration of GC agonists in cases of high risk of premature delivery (reviewed in Harris and Seckl^10^). We have previously shown that neurons and neural stem cells (NSCs) of rats prenatally exposed to high levels of the synthetic GC dexamethasone (Dex) exhibit a long-lasting increased susceptibility to oxidative stress.^11, 12^ Dex treatment *in vitro* decreases NSC proliferation, neuronal differentiation, and modifies the expression of genes associated with cellular senescence and mitochondrial functions in a GC receptor (GR)-mediated manner.^13^ The phenotypical alterations are associated with a decrease in total DNA methylation and the expression of DNA methyltransferases (DNMTs), and notably these global changes persists in 'daughter' NSCs never directly exposed to Dex, suggesting a *bona fide* epigenetic mechanism.^13^

DNA methylation is catalyzed and maintained by DNMTs (Dnmt1, Dnmt3a, and Dnmt3b).^14^ Dnmt1 and Dnmt3a are required for proper proliferation as well as neuronal and glial differentiation of NSCs.^15, 16, 17^ Genetic deletion of Dnmt3a leads to premature glial differentiation,^16, 18, 19^ and conditional knockout mice exhibit decreased adult neurogenesis.^17, 20, 21^ The understanding of the dynamic regulation of DNA methylation has increased significantly with the discovery of the ten-eleven translocation (Tet) family of methylcytosine dioxygenases (Tet1, Tet2, and Tet3). Tets catalyze the oxidation of 5-methylcytosine (5-mC) and generate 5-mC derivatives, including 5-hydroxymethylcytosine (5-hmC). Recent reports have demonstrated that deficiency of Tet1 is associated with impaired embryonic and adult neurogenesis^22, 23^ whereas overexpression of Tet1 impairs memory formation in mice.^24^ Tet3 is required for normal survival, proliferation, and differentiation of neural progenitor cells, but the mechanisms involved are not clarified.^25, 26^ Hence, a fine tuning of both Dnmts and Tets appears to be critical for the correct development and function of the brain.

In this study, we aimed at elucidating the mechanisms underlying the programming effects of the GC agonist Dex on the epigenome in cortical NSCs. By analyzing genome-wide DNA methylation with a MeDIP-like approach, we found a dramatic decrease in DNA methylation and identified numerous differentially methylated regions (DMRs) in Dex-exposed proliferating NSCs. The genome-wide Dex-induced changes in methylation were associated with a downregulation of Dnmt3a and an upregulation of Tet3 in both parent (P) NSCs and daughter (D) cells, which were never directly exposed to Dex. Interestingly, we found a similar global DNA hypomethylation along with Dnmt3a downregulation and Tet1–3 upregulation in the cerebral cortex of pups exposed to Dex *in utero*. We have previously identified Dickkopf 1 (Dkk1) as a direct target of Dex acting via GR binding to the Dkk1 promoter.^27^ Proteins of the Dkk family inhibit the canonical Wnt signaling and are essential for brain development.^28, 29^ We now show that the Dex-induced Dkk1 upregulation is heritable and dependent on Tet3 expression. Our results show that transient exposure to excess GC have dramatic and long-lasting effects on the epigenome of NSCs and specifically point to a critical Tet3-mediated dysregulation of Dnmt3a and Dkk1, both essential factors for proper forebrain development.

## Results

### Dex exposure induces persistent alterations in DNA methylation in NSCs

**Figure 1:**
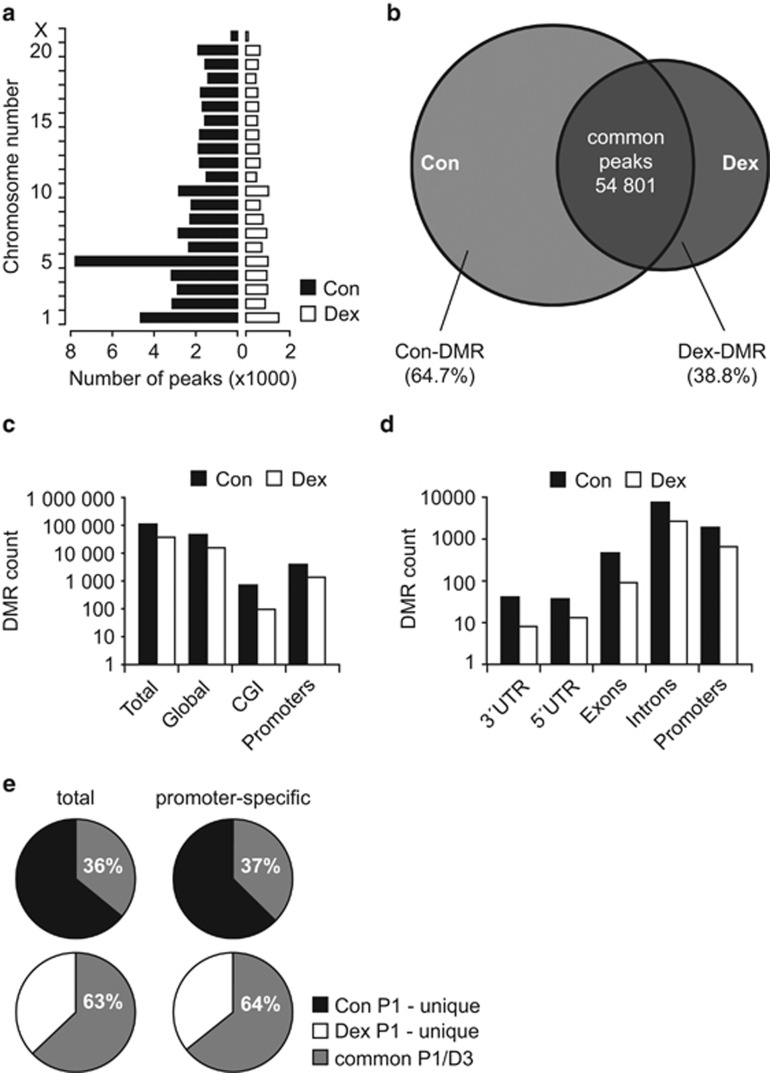
Overview of MBD sequencing results. (**a**) Chromosomal distribution of methylation peaks in control and Dex-exposed NSCs. (**b**) Venn diagram depicting the proportions of common and unique methylation peaks (DMRs) in control and Dex-exposed P1 NSCs. (**c**) The DMRs of control and Dex-exposed NSCs were normalized to refGene. Note that the genome-wide DNA hypomethylation induced by Dex exposure affects CpG islands (CGI) and promoter regions to a similar extent. (**d**) The DMRs distributed within CGI of refGene show similar distribution in control and Dex-exposed NSCs. (**e**) The proportion of methylation sites that is consistent across passages (P1 → D3) is higher in Dex exposed than in control NSCs (*P*<0.05, chi-square test for proportions)

To study the relationship between the response of NSCs to GC and chromatin modifications, we investigated the genome-wide DNA methylation in control and Dex-exposed NSCs derived from cortices of embryonic rats. We used a MeDIP-like approach in combination with high-throughput sequencing. Analysis by qPCR confirmed the separation of hypermethylated and non-methylated DNA in both control and Dex-exposed cells (Supplementary Figure S1). After sequencing, the prepared libraries were mapped onto the rat genome (Figure 1a). A range of 11 289 181–34 476 024 raw reads was generated and the PCR product bias was removed before mapping to the genome (Supplementary Table S3). The genome-wide DNA methylation levels were quantified by analyzing mapped sequencing reads as methylation peaks, using the supernatant of each sample as standard background, and are referred to as total number of peaks (Supplementary Table S4). The total number of methylation peaks corresponding to each chromosome in control and Dex-exposed NSCs are shown in Figure 1a.

Next, we removed the peaks shared by control and Dex-exposed cells, so that the remaining peaks were unique to each specific sample, and referred to as DMRs.^30, 31^ This yielded 110 000 DMRs in control, 37 005 DMRs in Dex-exposed NSCs, and 54 801 common methylation peaks (Figure 1b). To specifically characterize the changes in DNA methylation in NSCs, we deeply analyzed the DNA methylation pattern by dispersing the DMRs in different directions of genomic contexts normalizing with refGene. The overview of DMRs distribution related to refGene including CpG islands (CGI) and promoters showed a decrease in DMRs in Dex-exposed cells as compared to control cells (Figure 1c). Similar results were found when DMRs were distributed more specifically into promoters, exons, introns, and UTRs within CGI of refGene (Figure 1d).

We then analyzed the DNA methylation pattern in proliferating NSCs directly exposed to Dex ('parent', P1) and in cells that were never directly exposed ('daughter cells' after two passages from Dex exposure (D3). Dex-D3 cells showed increased genome-wide DNA methylation, also confirmed by the increase in 5-mC levels (Supplementary Figure S2). Interestingly, ~63% of the methylation peaks were preserved in Dex-D3 cells, as compared to only 36% in control D3 NSCs (Figure 1e), indicating that a large subset of Dex-induced alterations in genome-wide DNA methylation are stable across passages. We also identified hypermethylated genes whose promoters are located within the CGI in both P1 and D3 NSCs using GREAT ontology (GO) tools (http://www.geneontology.org) (Supplementary Tables S8 and S9). Interestingly, genes known to play a role in the regulation of cell proliferation, cell differentiation, migration, cellular senescence, DNA methylation, ion channels, mitochondrial function, and oxidative stress appeared among the ones differentially methylated (Supplementary Tables S10 and S11).

**Figure 2:**
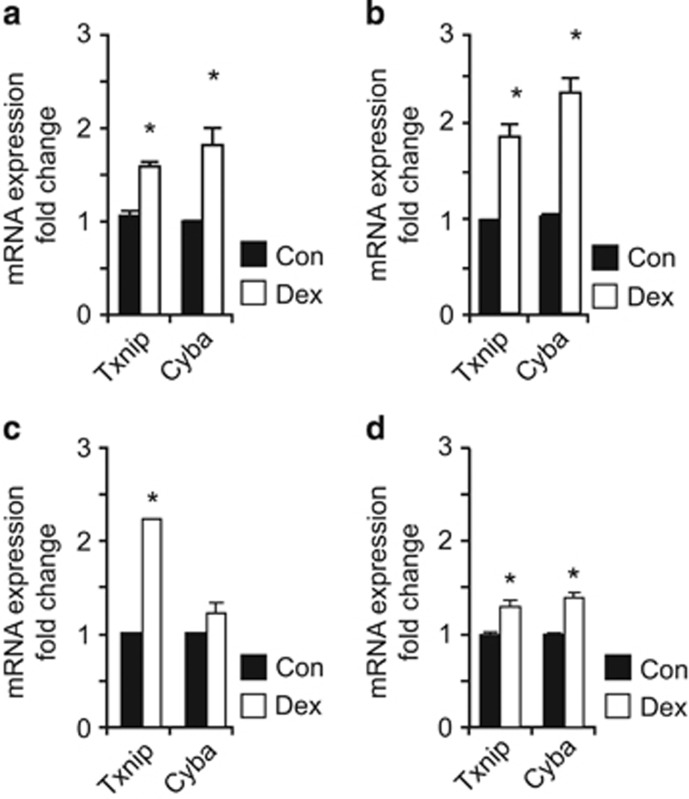
Single gene analysis of the DMR-enriched genes, Txnip and Cyba. Txnip and Cyba were upregulated in Dex exposed *versus* control in P1 NSCs (**a**), and the upregulation persists in D3 NSCs (**b**). (**c** and **d**) Similar pattern of mRNA expression regulation induced by exposure to Dex in differentiating NSCs (**c**) and *in vivo*, in the cortex of PND3 pups (**d**). The amount of target genes was normalized to *Hprt* and the relative increase was calculated as 2^*−ΔΔCr*^. Data presented as average±S.D. of at least three independent replicates. *P<0.05, Student's *t*-test

For further analyses, we selected relevant genes identified as DMR enriched, such as *Txnip* and *Cyba*, which are known to play a role in increased susceptibility to oxidative stress and mitochondrial function, and therefore of interest in relation to the Dex-induced phenotype of NSCs.^13, 27^ The mRNA expression of both genes was upregulated in Dex-exposed-proliferating NSCs (Figure 2a), in agreement with the promoter hypomethylation revealed by bisulfite conversion followed by MSP (Supplementary Figure S3). We then investigated whether the stability of the phenotype in D3 NSCs would correlate with changes in the expression of the selected genes. Both promoter methylation status (Supplementary Figure S3) and mRNA expression upregulation (Figure 2b) were persistent in Dex-exposed D3-proliferating NSCs, in line with our earlier report.^13^ Similar changes were also maintained in Dex-exposed differentiating NSCs (Figure 2c). To address the relevance of these findings *in vivo*, we analyzed the cortex of postnatal day 3 (PND3) mouse pups exposed to Dex *in utero* and detected an upregulation of both *Txnip* and *Cyba* mRNA expression (Figure 2d).

### Tet3 mediates the epigenetic effects of Dex and regulates Dnmt3a and Dkk1 expression

**Figure 3:**
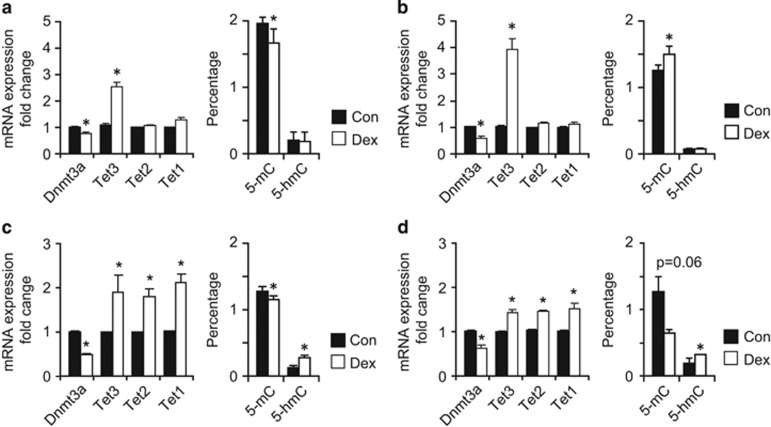
mRNA expression analysis for *Dnmt3a* and *Tet* family, and associated changes in the levels of 5-mC and 5-hmC. (**a** and **b**) Significant downregulation of *Dnmt3a* and upregulation of *Tet3*, but not *Tet1* and *2*, in both P1 (**a**) and D3 (**b**) proliferating NSCs. Dex exposure decreased 5-mC levels in P1 (**a**) and increased the 5-mC level in D3 (**b**) proliferating NSCs. The levels of 5-hmC are not altered by Dex exposure in either P1 or D3 cells. (**c**) Effects of exposure to Dex in spontaneously differentiating NSCs: *Dnmt3a* is downregulated, while *Tet1–3* are upregulated. In addition, 5-mC levels are decreased and 5-hmC levels are increased following Dex exposure in differentiating NSCs. (**d**) Similar gene regulation pattern found in the cortex of PND3 mouse pups exposed to Dex *in utero*: *Dnmt3a* downregulation and *Tet1–3* upregulation, associated with decreased 5-mC and increased 5-hmC levels. The amount of target genes was normalized to *Hprt* and the relative increase was calculated as 2^*−ΔΔCr*^. Data presented as average±S.D. of at least three independent replicates. *P<0.05, Student's *t*-test

To investigate the mechanisms underlying the persistence of a large proportion of methylation peaks induced by Dex, we focused our investigation on the genes mediating epigenetic effects. We found that *Tet3*, but not *Tet1* or *2*, was upregulated concomitantly with Dnmt3a downregulation in Dex-proliferating P1 NSCs (Figure 3a). This is consistent with DNA hypomethylation, and was confirmed by the decrease in 5-mC, while the level of 5-hmC were not significantly altered (Figure 3a). Proliferating daughter (D3) cells displayed a similar pattern of alterations in gene expression, but, intriguingly, this was associated with an increase in 5-mC levels (Figure 3b).

The *Dnmt3a* downregulation associated with *Tet3* upregulation was present also in differentiating NSCs exposed to Dex, and was accompanied by decreased 5-mC and increased 5-hmC levels (Figure 3c). In addition, *Tet1* and *2* were upregulated in differentiating NSCs exposed to Dex. The distinct pattern of gene expression regulation observed in differentiating NSCs was also present in the cortex of PND3 pups exposed to Dex *in utero* (Figure 3d), and this was associated with a decrease in 5-mC and an increase in 5-hmC levels. These results suggest that whereas an imbalance in *Dnmt3a* and *Tet3* expression was sufficient to alter 5-mC levels, alterations in global 5-hmC levels were dependent on a general upregulation of *Tet3* as well as *Tet1* and *Tet2*.

**Figure 4:**
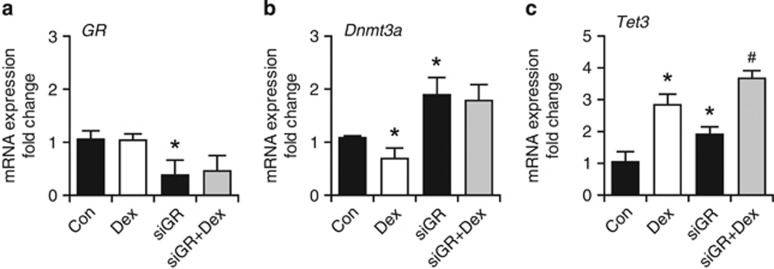
mRNA expression of *Dnmt3a* and *Tet3* is GR dependent. GR expression was knocked down by siRNA against GR (siGR). (**a**) Constitutive mRNA expression for *GR* was decreased by siGR and was not altered by Dex in the presence of siGR. The effect of Dex on *Dnmt3a* (**b**), but not *Tet3* (**c**), is abolished by siGR. The amount of target genes was normalized to *Hprt* and the relative increase was calculated as 2^*−ΔΔCr*^. Data presented as average±S.D. of at least three independent replicates. *P<0.05 *versus* Con (control), Student's *t*-test; ^#^*P*<0.05 *versus* siTet3, Student's *t*-test

**Figure 5:**
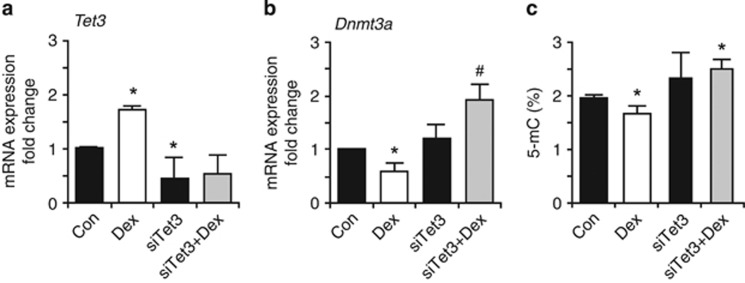
Tet3-dependent modulation of *Dnmt3a* expression and global DNA methylation. Tet3 expression was knocked down by siRNA against Tet3 (siTet3). (**a**) siTet3 reduced the constitutive expression of *Tet3* and abolished the upregulation induced by Dex. (**b**) The constitutive expression of *Dnmt3a* is not altered, but the Dex-induced downregulation is reversed by siTet3. (**c**) siTet3 does not alter global DNA methylation status in control cells, but the global DNA hypomethylation induced by Dex exposure is reversed. The amount of target genes was normalized to *Hprt* and the relative increase was calculated as 2^*−ΔΔCr*^. Data presented as average±S.D. of at least three independent replicates. *P<0.05 *versus* Con (control), Student's *t*-test; ^#^*P*<0.05 *versus* siTet3, Student's *t*-test

To investigate whether the observed effects were GR dependent, we performed siRNA-mediated knockdown of GR expression in P1-proliferating NSCs (Figure 4a). In contrast to the *Dnmt3a* downregulation, the Dex-induced upregulation of *Tet3* appeared not to be GR dependent (Figures 4b and c). The GR-mediated action may explain the direct effects of Dex on *Dnmt3a* observed in NSCs, however, it cannot account for the heritable changes in gene expression present in cells that were never directly exposed to Dex. To further investigate the role of Tet3, we knocked down its expression using siRNA interference in P1-proliferating NSCs (fold change 0.46±0.16; Figure 5a). Interestingly, Tet3 knockdown reversed the Dex-induced downregulation of *Dnmt3a* expression (Figure 5b) and increased 5-mC levels (Figure 5c).

**Figure 6:**
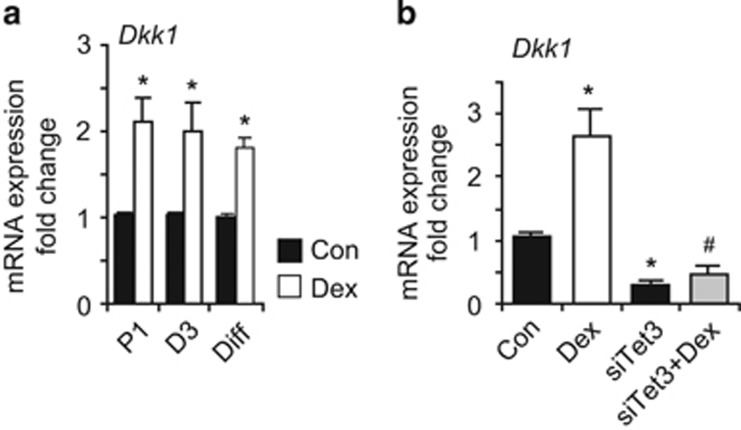
*Dkk1* mRNA expression regulation. (**a**) Exposure to Dex induces persistent upregulation of *Dkk1* in proliferating NSCs (P1 and D3), as well as in spontaneously differentiating NSCs (Diff). (**b**) Tet3 knockdown by siRNA (siTet3) reduced the constitutive expression and blocks the Dex-induced upregulation of *Dkk1*. The amount of target genes was normalized to *Hprt* and the relative increase was calculated as 2^*−ΔΔCr*^. Data presented as average±S.D. of at least three independent replicates. *P<0.05 *versus* Con (control), Student's *t*-test; ^#^*P*<0.05 *versus* siTet3, Student's *t*-test

Exposure to Dex alters the differentiation potential of NSCs and we have shown that Dkk1, which is upregulated in a GR-dependent manner (Supplementary Figure S4), is a critical mediator of the acute effects of Dex on the differentiation of human neural progenitors.^27^ *Dkk1* mRNA is upregulated even in D3-proliferating NSCs never directly exposed to Dex, as well as in differentiating NSCs (Figure 6a), suggesting that other mechanisms may be also involved. We then explored whether the expression of *Dkk1* was Tet3 dependent; Tet3 knockdown not only resulted in a downregulation of *Dkk1* in control NSCs, but also abolished the Dex-induced upregulation of Dkk1 in NSCs (Figure 6b). In summary, our results demonstrate that Tet3 is required for the stable Dex-induced alterations in DNA methylation as well as for *Dnmt3a* and *Dkk1* expression changes.

## Discussion

Here we have shown that GC induces strong and to a certain extent stable effects on DNA methylation in NSCs, and that Tet3 is an important regulator of Dnmt3a and Dkk1. The genome-wide DNA methylation analysis revealed a decrease in DMRs following Dex exposure in proliferating NSCs. The pattern of DNA methylation is very dynamic, and the methylation status of many gene promoters changes from P1 to D3. This is expected since global DNA methylation is dependent on the expression levels and activity of a large number of factors, including DNMTs, Tets, and DNA repair machinery. *In vivo*, Crudo *et al.*^32^ reported that the hypomethylation induced by acute administration of synthetic GC is not persistent, but is followed by hypermethylation of other gene promoters 14 days after the initial exposure. In line with these data, we also found that the Dex-induced pattern of global DNA methylation detected in P1 cells was partially reversed in D3 cells, which display increased global DNA methylation as compared to control D3 NSCs. Nevertheless, the pattern of gene promoter methylation in Dex-exposed-proliferating NSCs is rather stable, and we further focused on analyzing the mRNA expression of genes with stable changes in methylation peaks in the promoter region. Of relevance in relation to the Dex-induced NSC phenotype is that genes critical for the regulation of cell proliferation, differentiation, migration, senescence, DNA methylation, ion channels, mitochondrial function, and oxidative stress displayed stable promoter methylation in P1 and D3-proliferating NSCs. We show that the persistent upregulation of *Txnip*, which inhibits the reducing activity of thioredoxin, and *Cyba*, a NAD(P)H oxidase enzyme, in proliferating NSCs is associated with promoter hypomethylation. In addition, the expression of both *Txnip* and *Cyba* was upregulated in differentiating cells as well as in the postnatal mouse cortex. These data are consistent with the increased susceptibility to oxidative stress that we have previously reported after exposure to Dex.^11, 13^

Epigenetic regulation during development involves several key players.^22, 33^ The expression profiles of Tet family members vary depending on cell type and differentiation stage. In P1-proliferating NSCs, the absolute expression levels of Tet1 and 2 are very low, while in differentiating NSCs they are considerably higher. This variation in the expression may influence the detection of the Dex effects on individual Tets. The net effect is decreased 5-mC levels (i.e., global DNA hypomethylation), while the increase in 5-hmC levels becomes significant when not only *Tet3*, but also *Tet1* and *Tet2* are upregulated. This is in agreement with earlier reports on redundancy and compensatory actions of both Dnmts^34, 35^ and Tet family proteins^36, 37, 38, 39^ in accounting for global effects. Here, we show that *Dnmt3a* downregulation and *Tet3* upregulation are a persistent outcome of Dex exposure in NSCs, and that *Dnmt3a* upregulation is Tet3 dependent. Thus, if the GR dependence of *Dnmt3a* may explain the direct Dex effects observed in P1-proliferating NSCs, their persistence in D3 cells, as well as in differentiating cells and in postnatal mouse cortex can be attributed to Tet3.

Tet3 has been recognized as an important player in neuronal differentiation of neural progenitor cells, but the downstream mechanism remained elusive.^25, 26^ Here, we show that the role of Tet3 in neuronal differentiation is linked to its action on *Dkk1*. Dkk1 plays a critical role in regulating the terminal differentiation of neural progenitors during central nervous system development.^28, 29^ We reported earlier that Dex exposure reduces proliferations and neuronal differentiation in human neural progenitors by upregulating *DKK1* expression in a GR-dependent manner.^20^ The current findings suggest that the persistent effects of Dex on *Dkk1* upregulation are mediated by Tet3. Altogether, the persistent *Tet3* upregulation, which appears to be independent from GR activation, but accounts for the changes in *Dnmt3a* and *Dkk1* expression, plays a central role in the Dex-induced long-term effects in NSCs.

In conclusion, we provide evidence for a Tet3-dependent mechanism underlying the Dex-induced epigenetic reprogramming leading to heritable alterations of a fundamental player in cortical development. This mechanism may also play a role in the epigenetic programming occurring in response to early-life events that are linked to high levels of GC,^24, 25^ and may open new perspectives for preventive and therapeutic strategies.

## Materials and Methods

### Embryonic cortical NSC culture and exposure procedures

All experiments involving work with laboratory animals were approved by the local Animal Ethics Committee (Stockholm Northern Ethics Board of Animal Experimentation, ethical permission numbers: N79/08, N97/11, and N123/12). Primary cultures of NSCs were prepared as previously described.^11^ The cells were obtained from embryonic cortices (*n*=6–8 per cell preparation) from timed-pregnant Sprague-Dawley rats (Harlan Laboratories, Horst, The Netherlands) at E15 (the day of copulatory plug defined as E0) and dissected in HBSS (Life Technologies, Carlsbad, CA, USA). The tissue was mechanically dispersed, and meninges and larger cell clumps were allowed to sediment for 10 min. The cells were plated at a density of 40 000 per cm^2^ on a dish precoated with poly-l-ornithine and fibronectin (Sigma-Aldrich, Stockholm, Sweden). The cells were maintained in N-2 medium enriched with 10 ng/ml basic fibroblast growth factor (bFGF; R&D systems, Minneapolis, MN, USA) added every 24 h. The medium was changed every other day to keep cells in an undifferentiated and proliferative state. The cells were mechanically passaged via scraping in HBSS. Afterwards, the cells were gently mixed in N-2 medium, counted, and plated at the desired density. Under these culture conditions, NSC doubling time was ~20 h. To investigate the heritable effects of Dex on proliferating NSCs, we exposed NSCs to Dex (1 *μ*M) for 48 h as described earlier.^13^ Parental (P1) cells were harvested at the end of the exposure to Dex. We then passaged the cells in the presence of bFGF, but without adding Dex to the culture medium to obtain daughter cells (D2 and D3). Mitotically heritable effects were investigated in D3 cells that had never been exposed to Dex, 72 h after respective passaging.

### Immunocytochemistry

NSCs were fixed in 4% PFA for 1 h at 4°C, followed by washing in PBS. The NSCs were incubated with mouse anti-tubulin III (Tuj1, 1 : 400; Covance, Princeton, NJ, USA) and rabbit anti-glial fibrillary acidic protein (GFAP, 1 : 800; DakoCytomation, Glostrup, Denmark) diluted in PBS containing 0.3% Triton X-100 and 0.5% bovine serum albumin (BSA; Boehringer Mannheim, Mannheim, Germany) overnight in a humid chamber at 4°C. The cells were then rinsed with PBS and incubated with an appropriate secondary FITC-conjugated antibody for 1 h at RT (1 : 200; Alexa, Invitrogen, Carlsbad, CA, USA). Cell nuclei were counterstained with Hoechst 33342 (Sigma-Aldrich) (1 mg/ml) for 5 min. After rinsing with PBS, the coverslips were mounted onto slides with VECTASHIELD mounting medium (Vector Laboratories, Inc., Burlingame, CA, USA). The cells were examined using fluorescence microscope (Nikon Eclipse Ti-S, BergmanLabora AB, Danderyd, Sweden) and images were captured using Nikon camera (Nikon Digital Sight DS-Qi1MC, BergmanLabora AB). All experiments were performed in triplicates and repeated three times. Semiquantitative analyses were performed by counting at least 100 cells per coverslip in triplicates.

### siRNA-mediated knockdown

siRNA specific to rat GR was delivered using a Nucleofector kit according to the manufacturer's instructions (Amaxa, Lonza, Basel, Switzerland). Nucleofected NSCs were transferred into a 6-cm dish containing warm N-2 medium. At 3 h after neucleofection, NSCs were treated with 1 *μ*M Dex for 24 h.

A Smartpool mix of four siRNA-targeting rat Tet3 and negative siRNA control were purchased from Dharmacon (Thermo Scientific, Stockholm, Sweden). NSCs were grown in N-2 medium with 10 ng/ml bFGF for 24 h. Then N-2 medium were replaced with a Smartpool mix and media supplement. The cells were incubated with Smartpool mix for 72 h in the presence of bFGF and treated with 1 *μ*M Dex for the last 24 h. Then cells were harvested for global DNA methylation and gene expression analysis. Knockdown efficiency was analyzed by quantitative rtPCR. All experiments were performed in triplicates and repeated at least three times.

### *In vivo* experiments

We investigated the long-lasting effects of Dex in mouse pups. To this end, we injected pregnant female C57Bl/6 mice with Dex dissolved in sterile saline (0.05 mg/kg/day; injection volume 10 ml/kg) from gestational day (GD) 14 until delivery (GD19–20). The control females were injected with an equivalent volume of sterile saline. On PND3, the male offspring were killed by decapitation and the brain was rapidly dissected on ice and stored at −80°C until processing. Next, RNA and DNA were extracted from dissected cortices using RNA and DNA extraction kits as instructed by the manufacturer. RNA was used for the analysis of gene expression by qPCR, while the DNA was used for measuring global DNA methylation and hydroxymethylation as described below. PCR primer sequences for analysis of mice tissues are available in Supplementary Table S1.

### Extraction of total RNA, cDNA synthesis, and real-time PCR

Total RNA was isolated from NSCs using the RNeasy Mini kit (Qiagen, VWR, Stockholm, Sweden). Integrity and concentration of extracted RNA was measured by NanoDrop 1000 spectrophotometer (Thermo Scientific, Wilmington, DE, USA). cDNA was prepared using 1 *μ*g total RNA and 0.5 *μ*g of oligo-dT primer following the instructions of Superscript II first-strand cDNA synthesis kit (Invitrogen). Amplification reactions were performed with 1 *μ*l cDNA, SYBR Green mix (Applied Biosystems, Stockholm, Sweden) and 0.2 *μ*M of forward and reverse primers. The reaction volume was adjusted to 25 *μ*l with DEPC water. Negative control reactions contained water instead of cDNA as template. Quantitative real-time PCR was performed using an ABI Prism 7000 Sequence Detection System with SDS version 2.1 software (Applied Biosystems). The PCR cycle conditions were 50°C for 2 min, 95°C for 10 min, 95°C for 15 s, and 60°C for 1 min (40 cycles). To evaluate the amplification of a specific sample, final melting curve (from 60°C up to 95°C) was added under continuous fluorescence measurements. All expression values were normalized against the housekeeping gene HPRT (ΔCT=CTtarget gene−CTHPRT). Relative expression levels were calculated as ΔΔCT=ΔCTDex–ΔCTcontrol and relative expression changes were calculated as 2-ΔΔCT. Representative values are shown as mean±S.E.M. All experiments were performed in triplicates and repeated at least three times. PCR primer sequences for analysis of NSCs are available in Supplementary Table S1.

### Genomic DNA extraction

DNA was prepared using the XL GenDNA extraction module kit (Diagenode, Liège, Belgium) according to the manufacturer's instructions. Quality and quantity of DNA was measured using NanoDrop spectrophotometer (Thermo Scientific) and Quant-iT PicoGreen dsDNA reagent and kits (Invitrogen).

### DNA methylation and hydroxymethylation assay

DNA was prepared using the GeneElute mammalian genomic DNA miniprep kit (Sigma-Aldrich) according to the manufacturer's instructions. DNA quality and concentration was measured by NanoDrop 1000 spectrophotometer (Thermo Scientific). Separately global DNA methylation (5-mC) and hydroxymethylation (5-hmC) were determined using two different quantification kits (Epigentek, New York, NY, USA) as instructed by the manufacturer.

### Methyl-DNA immunoprecipitation sequencing, MBD-seq

DNA was sonicated using Bioruptor 200 (Diagenode) at high frequency with 30 s off/on cycles. The average length of sonicated DNA was 200 bp, which was determined by the gel electrophoresis. We used 1.2 *μ*g of sonicated DNA for subsequent MBD2 enrichment using MethylMiner methylated DNA enrichment kit (Life Technologies). Briefly, first 10 *μ*l of Dynabeads (Life Technologies) M-280 streptavidin were cleaned by 1 × bind/wash buffer and 3.5 *μ*g of BMD-biotin protein was mixed with clean Dynabeads (Life Technologies) on a rotating mixer for 1 h. Then DNA fragments were incubated with the coupled MBD-beads overnight at 4°C. After removing non-captured DNA as supernatant, captured DNA was isolated by NaCl gradient elution (0.5 and 1 M). The accuracy of the assay was confirmed by using kit-supplied control DNA. Isolation of hypermethylated (0.5 and 1 M) and non-methylated DNAs (supernatant) were confirmed by quantitative real-time PCR analysis using Tsh2b (methylation-specific primer) or Gapdh (non-methylation-specific primer) and those primer sequences were bought from Diagenode. The recovered DNA was quantified by Qubit (Invitrogen) and 50 ng of immunoprecipited DNA was used for library preparation using a kit from New England Biolabs (NEB# E6240S/L, BioNordika Sweden AB, Stockholm, Sweden). Subsequently, the library was analyzed by HiSeq 2000, Illumina, Inc (San Diego, CA, USA). The sequence tags were then aligned to the rat genome (assembly rn4) with the Bowtie alignment tool (http://bowtie-bio.sourceforge.net/index.shtml). To avoid any PCR bias, we allowed only one read per chromosomal position. Next, the peaks (hypermethylated regions) were identified using MACS software^40, 41^ and the rat CGIs were downloaded from the UCSC database (http://genome.ucsc.edu).

The genome-wide DNA methylation levels were quantified by analyzing mapped sequencing reads as methylation peaks, using the supernatant of each sample as standard background and are referred to as total number of peaks (Supplementary Table S4).

### Statistical analysis

One-way analysis of variance (ANOVA) followed by Bonferroni's *post hoc* test was performed. Student's *t*-test was used for comparisons of two groups. The significance value was set at *P*<0.05.

## Abbreviations

11*β*HSD2: 11 *β*-hydroxysteroid dehydrogenase 2
5-hmC: 5-hydroxymethylcytosine
5-mC: 5-methylcytosine
CGI: CpG islands (DNA regions enriched in CpG sequences)
CpG: cytosine-phosphate-guanine (DNA sequence)
Cyba: cytochrome b-245, alpha polypeptide
Dex: dexamethasone (synthetic GC analog)
Dkk1: dickkopf 1
DMR: differentially methylated region
DNMT: DNA methyltransferase
FGF: fibroblast growth factor
GC: glucocorticoid (hormone)
GR: glucocorticoid receptor
HBSS: Hank's balanced salt solution
HPRT: hypoxanthine-guanine phosphoribosyltransferase
MeDIP: methylated DNA immunoprecipitation
MSP: methylation-specific PCR
NSC: neural stem cell
PCR: polymerase chain reaction
Tet: ten-eleven translocation methylcytosine dioxygenase
Txnip: thioredoxin inhibiting protein

## References

[bib1] Fukumoto K, Morita T, Mayanagi T, Tanokashira D, Yoshida T, Sakai A et al. Detrimental effects of glucocorticoids on neuronal migration during brain development. Mol Psychiatry 2009; 14: 1119–1131. 19564873 10.1038/mp.2009.60

[bib2] Lemaire V, Koehl M, Le Moal M, Abrous DN. Prenatal stress produces learning deficits associated with an inhibition of neurogenesis in the hippocampus. Proc Natl Acad Sci USA 2000; 97: 11032–11037. 11005874 10.1073/pnas.97.20.11032 PMC27143

[bib3] Lucassen PJ, Bosch OJ, Jousma E, Krömer S, Andrew R, Seckl JR et al. Prenatal stress reduces postnatal neurogenesis in rats selectively bred for high, but not low, anxiety: possible key role of placental 11beta-hydroxysteroid dehydrogenase type 2. Eur J Neurosci 2009; 29: 97–103. 19032587 10.1111/j.1460-9568.2008.06543.x

[bib4] Weinstock M. The long-term behavioural consequences of prenatal stress. Neurosci Biobehav Rev 2008; 32: 1073–1086. 18423592 10.1016/j.neubiorev.2008.03.002

[bib5] Matthews SG, Owen D, Kalabis G, Banjanin S, Setiawan EB, Dunn EA et al. Fetal glucocorticoid exposure and hypothalamo-pituitary-adrenal (HPA) function after birth. Endocr Res 2004; 30: 827–836. 15666833 10.1081/erc-200044091

[bib6] Wong EYH, Herbert J. Raised circulating corticosterone inhibits neuronal differentiation of progenitor cells in the adult hippocampus. Neuroscience 2006; 137: 83–92. 16289354 10.1016/j.neuroscience.2005.08.073 PMC2651634

[bib7] Gould E, Tanapat P, McEwen BS, Flügge G, Fuchs E. Proliferation of granule cell precursors in the dentate gyrus of adult monkeys is diminished by stress. Proc Natl Acad Sci USA 1998; 95: 3168–3171. 9501234 10.1073/pnas.95.6.3168 PMC19713

[bib8] Fenoglio K, Brunson KL, Baram TZ. Hippocampal neuroplasticity induced by early-life stress: functional and molecular aspects. Front Neuroendocrinol 2006; 27: 180–192. 16603235 10.1016/j.yfrne.2006.02.001 PMC2937188

[bib9] Benediktsson R, Calder AA, Edwards CR, Seckl JR. Placental 11 beta-hydroxysteroid dehydrogenase: a key regulator of fetal glucocorticoid exposure. Clin Endocrinol (Oxf) 1997; 46: 161–166. 9135697 10.1046/j.1365-2265.1997.1230939.x

[bib10] Harris A, Seckl J. Glucocorticoids prenatal stress and the programming of disease. Horm Behav 2011; 59: 279–289. 20591431 10.1016/j.yhbeh.2010.06.007

[bib11] Ahlbom E, Gogvadze V, Chen M, Celsi G, Ceccatelli S. Prenatal exposure to high levels of glucocorticoids increases the susceptibility of cerebellar granule cells to oxidative stress-induced cell death. Proc Natl Acad Sci USA 2000; 97: 14726–14730. 11114198 10.1073/pnas.260501697 PMC18986

[bib12] Ceccatelli S, Tamm C, Zhang Q, Chen M. Mechanisms and modulation of neural cell damage induced by oxidative stress. Physiol Behav 2007; 92: 87–92. 17628619 10.1016/j.physbeh.2007.05.048

[bib13] Bose R, Moors M, Tofighi R, Cascante A, Hermanson O, Ceccatelli S. Glucocorticoids induce long-lasting effects in neural stem cells resulting in senescence-related alterations. Cell Death Dis 2010; 1: e92. 21368868 10.1038/cddis.2010.60 PMC3032322

[bib14] Bestor TH. The DNA methyltransferases of mammals. Hum Mol Genet 2000; 9: 2395–2402. 11005794 10.1093/hmg/9.16.2395

[bib15] Feng J, Chang H, Li E, Fan G. Dynamic expression of de novo DNA methyltransferases Dnmt3a and Dnmt3b in the central nervous system. J Neurosci Res 2005; 79: 734–746. 15672446 10.1002/jnr.20404

[bib16] Wu H, Coskun V, Tao J, Xie W, Ge W, Yoshikawa K et al. Dnmt3a-dependent nonpromoter DNA methylation facilitates transcription of neurogenic genes. Science 2010; 329: 444–448. 20651149 10.1126/science.1190485 PMC3539760

[bib17] Hutnick LK, Golshani P, Namihira M, Xue Z, Matynia A, Yang XW et al. DNA hypomethylation restricted to the murine forebrain induces cortical degeneration and impairs postnatal neuronal maturation. Hum Mol Genet 2009; 18: 2875–2888. 19433415 10.1093/hmg/ddp222 PMC2706688

[bib18] Fan G, Martinowich K, Chin MH, He F, Fouse SD, Hutnick L et al. DNA methylation controls the timing of astrogliogenesis through regulation of JAK-STAT signaling. Development 2005; 132: 3345–3356. 16014513 10.1242/dev.01912

[bib19] Wu Z, Huang K, Yu J, Le T, Namihira M, Liu Y et al. Dnmt3a regulates both proliferation and differentiation of mouse neural stem cells. J Neurosci Res 2012; 90: 1883–1891. 22714992 10.1002/jnr.23077 PMC3418436

[bib20] LaPlant Q, Vialou V, Covington HE 3rd, Dumitriu D, Feng J, Warren BL et al. Dnmt3a regulates emotional behavior and spine plasticity in the nucleus accumbens. Nat Neurosci 2010; 13: 1137–1143. 20729844 10.1038/nn.2619 PMC2928863

[bib21] Nguyen S, Meletis K, Fu D, Jhaveri S, Jaenisch R. Ablation of de novo DNA methyltransferase Dnmt3a in the nervous system leads to neuromuscular defects and shortened lifespan. Dev Dyn 2007; 236: 1663–1676. 17477386 10.1002/dvdy.21176

[bib22] Hahn MA, Qiu R, Wu X, Li AX, Zhang H, Wang J et al. Dynamics of 5-hydroxymethylcytosine and chromatin marks in Mammalian neurogenesis. Cell Rep 2013; 3: 291–300. 23403289 10.1016/j.celrep.2013.01.011 PMC3582786

[bib23] Zhang R-R, Cui Q-Y, Murai K, Lim YC, Smith ZD, Jin S et al. Tet1 regulates adult hippocampal neurogenesis and cognition. Cell Stem Cell 2013; 13: 237–245. 23770080 10.1016/j.stem.2013.05.006 PMC4474382

[bib24] Kaas GA, Zhong C, Eason DE, Ross DL, Vachhani RV, Ming G-LL et al. TET1 controls CNS 5-methylcytosine hydroxylation, active DNA demethylation, gene transcription, and memory formation. Neuron 2013; 79: 1086–1093. 24050399 10.1016/j.neuron.2013.08.032 PMC3816951

[bib25] Li T, Yang D, Li J, Tang Y, Yang J, Le W. Critical role of Tet3 in neural progenitor cell maintenance and terminal differentiation. Mol Neurobiol 2014; 51: 142–154. 24838624 10.1007/s12035-014-8734-5

[bib26] Lv X, Jiang H, Liu Y, Lei X, Jiao J. MicroRNA-15b promotes neurogenesis and inhibits neural progenitor proliferation by directly repressing TET3 during early neocortical development. EMBO Rep 2014; 15: 1305–1315. 25344561 10.15252/embr.201438923 PMC4264933

[bib27] Moors M, Bose R, Johansson-Haque K, Edoff K, Okret S, Ceccatelli S. Dickkopf 1 mediates glucocorticoid-induced changes in human neural progenitor cell proliferation and differentiation. Toxicol Sci 2012; 125: 488–495. 22048647 10.1093/toxsci/kfr304

[bib28] Munji RN, Choe Y, Li G, Siegenthaler JA, Pleasure SJ. Wnt signaling regulates neuronal differentiation of cortical intermediate progenitors. J Neurosci 2011; 31: 1676–1687. 21289176 10.1523/JNEUROSCI.5404-10.2011 PMC3040956

[bib29] Diep DB, Hoen N, Backman M, Machon O, Krauss S. Characterisation of the Wnt antagonists and their response to conditionally activated Wnt signalling in the developing mouse forebrain. Brain Res Dev Brain Res 2004; 153: 261–270. 15527894 10.1016/j.devbrainres.2004.09.008

[bib30] Yu W, Jin C, Lou X, Han X, Li L, He Y et al. Global analysis of DNA methylation by methyl-capture sequencing reveals epigenetic control of cisplatin resistance in ovarian cancer cell. PLoS One 2011; 6: e29450. 22216282 10.1371/journal.pone.0029450 PMC3245283

[bib31] Zhao Y, Guo S, Sun J, Huang Z, Zhu T, Zhang H et al. Methylcap-seq reveals novel DNA methylation markers for the diagnosis and recurrence prediction of bladder cancer in a Chinese population. PLoS One 2012; 7: e35175. 22529986 10.1371/journal.pone.0035175 PMC3328468

[bib32] Crudo A, Suderman M, Moisiadis VG, Petropoulos S, Kostaki A, Hallett M et al. Glucocorticoid programming of the fetal male hippocampal epigenome. Endocrinology 2013; 154: 1168–1180. 23389956 10.1210/en.2012-1980

[bib33] Wang T, Pan Q, Lin L, Szulwach KE, Song C-XX, He C et al. Genome-wide DNA hydroxymethylation changes are associated with neurodevelopmental genes in the developing human cerebellum. Hum Mol Genet 2012; 21: 5500–5510. 23042784 10.1093/hmg/dds394 PMC3516134

[bib34] Challen GA, Sun D, Mayle A, Jeong M, Luo M, Rodriguez B et al. Dnmt3a and Dnmt3b have overlapping and distinct functions in hematopoietic stem cells. Cell Stem Cell 2014; 15: 350–364. 25130491 10.1016/j.stem.2014.06.018 PMC4163922

[bib35] Feng J, Zhou Y, Campbell SL, Le T, Li E, Sweatt JD et al. Dnmt1 and Dnmt3a maintain DNA methylation and regulate synaptic function in adult forebrain neurons. Nat Neurosci 2010; 13: 423–430. 20228804 10.1038/nn.2514 PMC3060772

[bib36] Koh KP, Yabuuchi A, Rao S, Huang Y, Cunniff K, Nardone J et al. Tet1 and Tet2 regulate 5-hydroxymethylcytosine production and cell lineage specification in mouse embryonic stem cells. Cell Stem Cell 2011; 8: 200–213. 21295276 10.1016/j.stem.2011.01.008 PMC3134318

[bib37] Dawlaty MM, Breiling A, Le T, Raddatz G, Barrasa MI, Cheng AW et al. Combined deficiency of Tet1 and Tet2 causes epigenetic abnormalities but is compatible with postnatal development. Dev Cell 2013; 24: 310–323. 23352810 10.1016/j.devcel.2012.12.015 PMC3574201

[bib38] Dawlaty MM, Ganz K, Powell BE, Hu Y-C, Markoulaki S, Cheng AW et al. Tet1 is dispensable for maintaining pluripotency and its loss is compatible with embryonic and postnatal development. Cell Stem Cell 2011; 9: 166–175. 21816367 10.1016/j.stem.2011.07.010 PMC3154739

[bib39] Dawlaty MM, Breiling A, Le T, Barrasa MI, Raddatz G, Gao Q et al. Loss of Tet enzymes compromises proper differentiation of embryonic stem cells. Dev Cell 2014; 29: 102–111. 24735881 10.1016/j.devcel.2014.03.003 PMC4035811

[bib40] Feng J, Liu T, Zhang Y. Using MACS to identify peaks from ChIP-Seq data. Curr Protoc Bioinformatics 2011; Chapter 2 Unit 2.14. 21633945 10.1002/0471250953.bi0214s34 PMC3120977

[bib41] Zhang Y, Liu T, Meyer CA, Eeckhoute J, Johnson DS, Bernstein BE et al. Model-based analysis of ChIP-Seq (MACS). Genome Biol 2008; 9: R137. 18798982 10.1186/gb-2008-9-9-r137 PMC2592715

