# Supplementary material for: Tet3 mediates stable glucocorticoid-induced alterations in DNA methylation and Dnmt3a/Dkk1 expression in neural progenitors
Source: Cell Death Dis. 2015 Jun 18;6(6):e1793–. doi: 10.1038/cddis.2015.159 (PMC4669838; doi:10.1038/cddis.2015.159)
Supplement: Supplementary Information [file cddis2015159x1.doc]

**Supplementary information:**

***Supplementary Materials and Methods***

*Methylation-specific PCR (MSP)*

To validate the methylation status of the selected regions, we used methylation specific PCR (MSP) in control and Dex exposed NSCs. MSP was used for gene promoter methylation as described elsewhere ^1^. Briefly, DNA samples were treated by bisulfite for conversion of unmethylated cytosine to uracil but not methylated cytosine using a MethylCode™ Bisulfite Conversion Kit (Invitrogen). Primers were designed using Methprimer program ^2^ that gave two different set of primers for the methylated and unmethylated sequences(Table S2).Next, bisulfite-treated DNA was amplified by MSP using EpiMark Hot Start Taq DNA Polymerase (New England Biolabs) according to manufacturer’s protocol. After amplification, MSP products were visualized on an E-gel 2% with SyBR Safe (Invitrogen) and methylated and unmethylated bands were observed in control versus treatment.

**References**

1 Herman JG, Graff JR, Myöhänen S, Nelkin BD, Baylin SB, Myohanen S. Methylation-specific PCR: a novel PCR assay for methylation status of CpG islands. *Proc Natl Acad Sci U S A* 1996; **93**: 9821–9826.

2 Li LC, Dahiya R. MethPrimer: designing primers for methylation PCRs. *Bioinformatics* 2002; **18**: 1427–1431.

***Supplementary Figures and tables***

**Fig. S1** Analysis of methylated DNA enrichment by qPCR. Methylated DNA fragments were immunoprecipitated using a MeDIP like approach. The methylated (0.5M and 1M) and unmethylated (supernatant), and control (input) DNA fragments were amplified with primer pairs specific for TSH2B and GAPDH genomic regions (TSH2B primer pair corresponds to genomic locus that is known as highly methylated in all somatic cells and GAPDH primer pair for unmethylated control). qPCR analysis reveals the ratio of enrichment of methylated and unmethylated DNA fragments compared to control (input).

**Fig. S2** Distribution and characterization of DNA methylation in chromosomes and gene regions. (A) Chromosome distribution of methylation peaks. (B) Common and unique methylation peaks in D3 NSCs.

**Fig. S3** Confirmation of promoter methylation status by bisulfite conversion followed by methylation-specific PCR (MSP). MSP primers were designed in the promoter of Txnip and Cyba, specific regions for either methylated or unmethylated DNA amplification. Note that the signal for unmethylated DNA is stronger in Dex-exposed NSCs than in controls in both promoters investigated in P1 and D3.

**Fig. S4** The mRNA expression of *Dkk1* is regulated by both GR and promoter methylation. (A) GR knockdown down-regulates *Dkk1* expression and blocks the Dex-induced up-regulation. (B) Dex exposure results in Dkk1 promoter hypomethylation as validated by bisulfite conversion followed by MSP. The amount of target genes was normalized to *Hprt* and the relative increase was calculated as $2^{-\Delta\Delta C_{T}}$. *p<0.05, student’s t-test.

**Table S1** List of primer sequences for mRNA expression analysis by qPCR.

**Table S2** List of primer sequences targeting promoter regions for DNa methylation analysis by MSP.

**Table S3** Number of reads generated by MeDIP-like approach for each sample. Total reads, uniquely mapped reads and distinct read positions in the genome.

**Table S4** Methylated DNA fragments identified by MACS in control P1 NSCs. Tag size: 50; model fold: 10; p value cutoff: 1e-5.

**Table S5** Methylated DNA fragments identified by MACS in Dex-exposed P1 NSCs. Tag size: 50; model fold: 10; p value cutoff: 1e-5.

**Table S6** Methylated DNA fragments identified by MACS in control D3 NSCs. Tag size: 50; model fold: 10; p value cutoff: 1e-5.

**Table S7** Methylated DNA fragments identified by MACS in Dex-exposed D3 NSCs. Tag size: 50; model fold: 10; p value cutoff: 1e-5.

**Table S8** Analysis of differential methylation level of gene regions in P1 NSCs. Gene, gene region, binomial false discovery rate (FDR), p-value and fold enrichment.

**Table S9** Analysis of differential metylation level of gene regions in D3 NSCs. Gene, gene region, binomial false discovery rate (FDR), p-value and fold enrichment.

**Table S10** GREAT ontology enrichments of methylated DNA fragments in P1 NSCs.

**Table S11** GREAT ontology enrichments of methylated DNA fragments in D3 NSCs.
