# Supplementary figures and images for: Tet3 mediates stable glucocorticoid-induced alterations in DNA methylation and Dnmt3a/Dkk1 expression in neural progenitors
Source: Cell Death Dis. 2015 Jun 18;6(6):e1793–. doi: 10.1038/cddis.2015.159 (PMC4669838; doi:10.1038/cddis.2015.159)

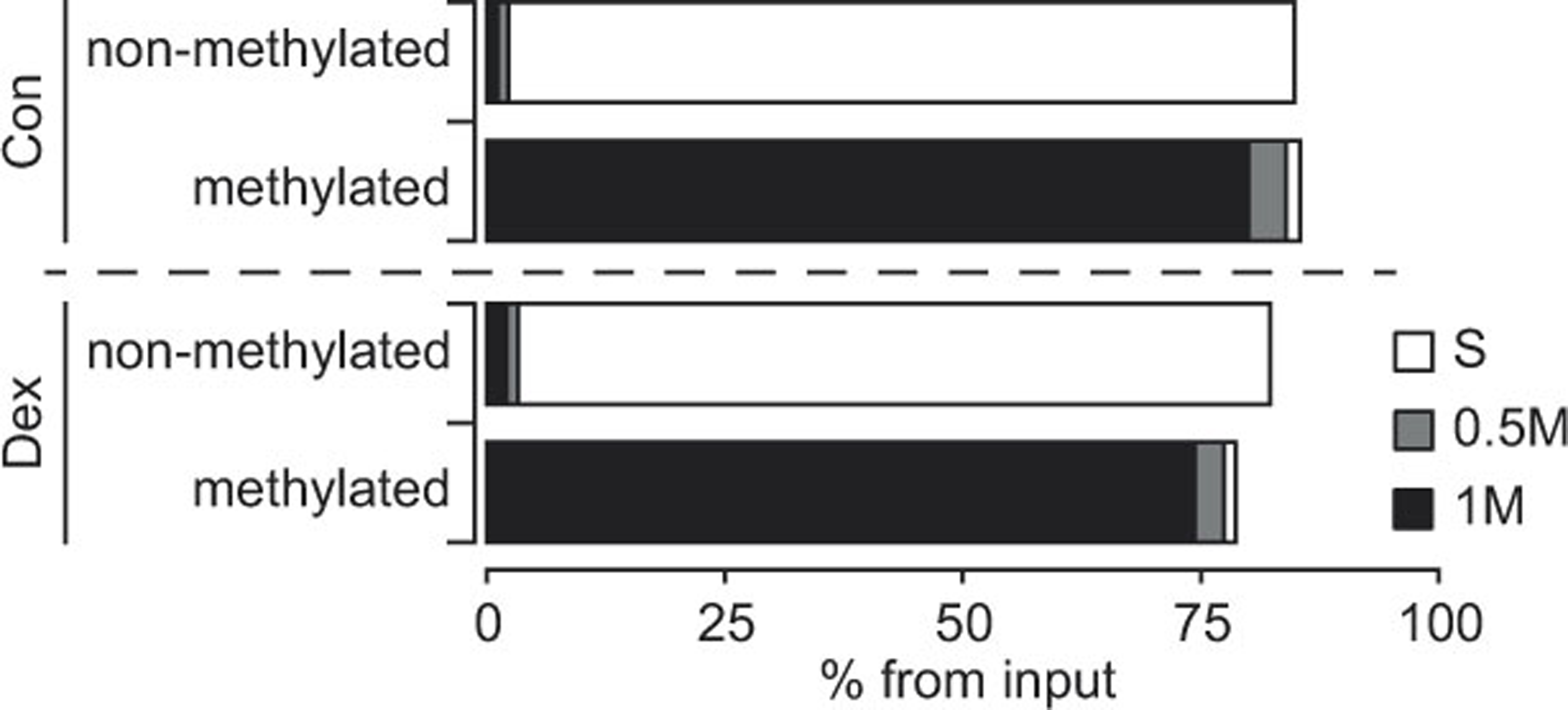

Supplement: Supplementary Figure 1 [file cddis2015159x2.tif]

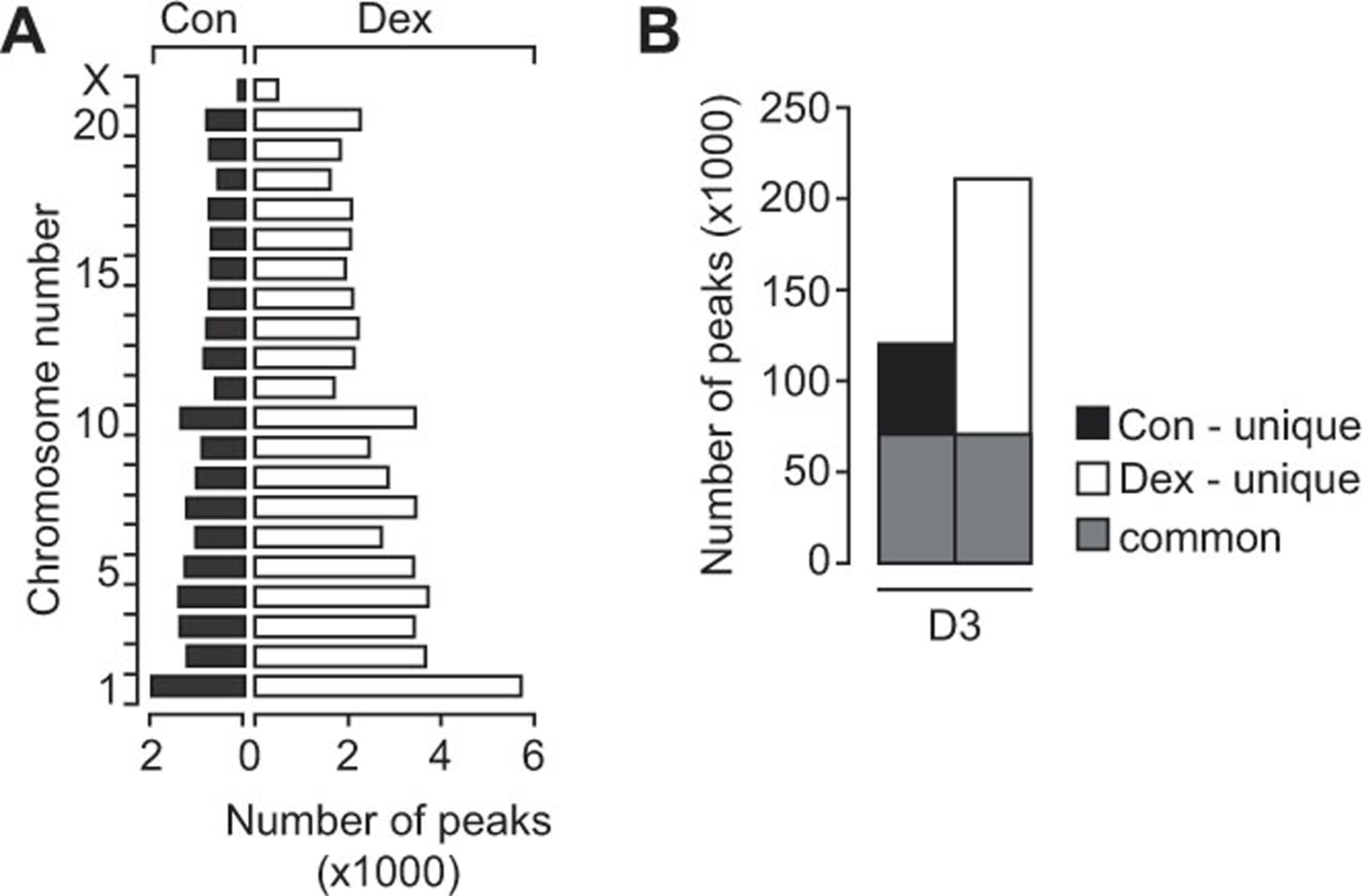

Supplement: Supplementary Figure 2 [file cddis2015159x3.tif]

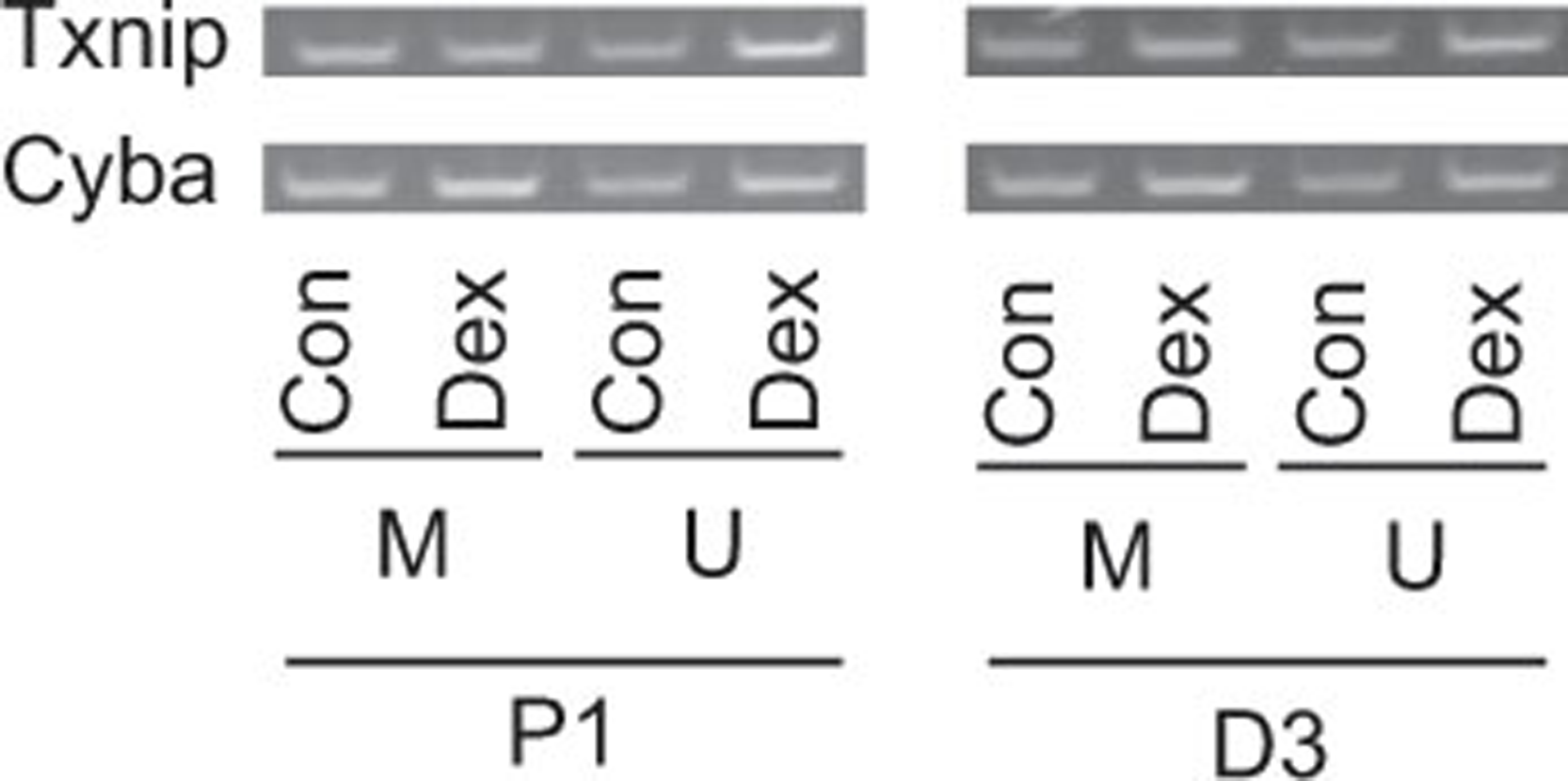

Supplement: Supplementary Figure 3 [file cddis2015159x4.tif]

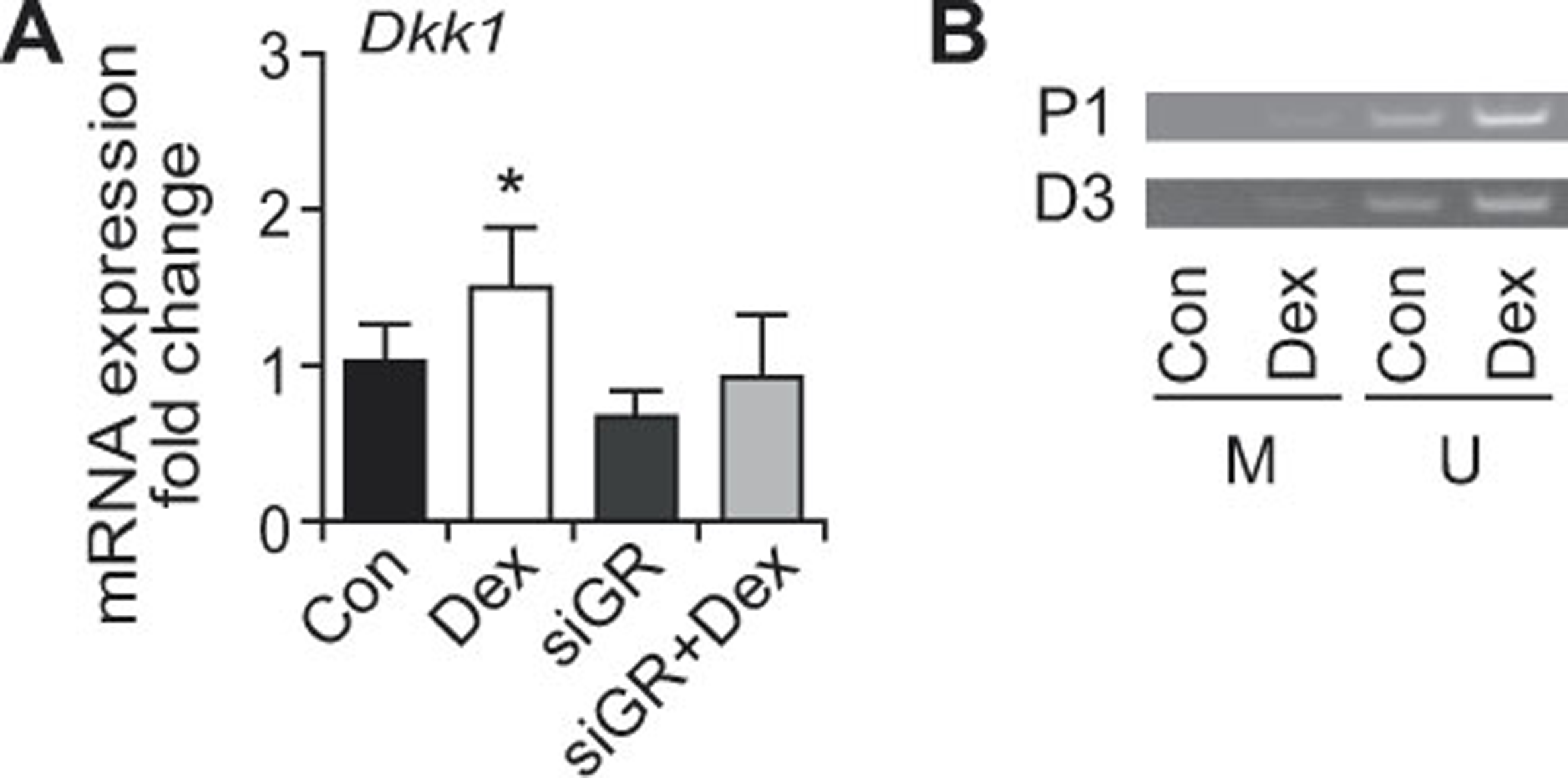

Supplement: Supplementary Figure 4 [file cddis2015159x5.tif]
