# Supplementary material for: Tet3 mediates stable glucocorticoid-induced alterations in DNA methylation and Dnmt3a/Dkk1 expression in neural progenitors
Source: Cell Death Dis. 2015 Jun 18;6(6):e1793–. doi: 10.1038/cddis.2015.159 (PMC4669838; doi:10.1038/cddis.2015.159)
Supplement: Supplementary Table 1 [file cddis2015159x6.doc]

**Table S1** qPCR primer sequences

| **Primer name** | species | forward | reverse |
| --- | --- | --- | --- |
| **Dkk1** | mouse | gctcatctcggagaaagtcg | catgatccagaaagccacct |
| **Dkk3** | mouse | tgtttgaagggagaggatgg | ttgtgtagccactgcctcag |
| **Txnip** | mouse | catgaggcctggaaacaaat | actggtgccattaggtcagg |
| **Cyba** | mouse | gtggactcccattgagccta | ctcctcttcaccctcactcg |
| **Dmnt1** | mouse | ccaccaccaagctggtctat | tgccaccaaacttcaccata |
| **Dnmt3a** | mouse | acttggagaagcggagtgaa | ctgttctttgccctctcctg |
| **Dnmt3b** | mouse | acttggtgattggtggaagc | ccagaagaatggacggttgt |
| **Tet1** | mouse | gcgtgaagctcaaacatcaa | gtgcagcttcctgttccttc |
| **Tet2** | mouse | gttctcaacgagcaggaagg | tgagatgcggtactctgcac |
| **Tet3** | mouse | tccggattgagaaggtcatc | ccaggccaggatcaagataa |
| **Mtr** | mouse | ttgcaactccattgcttctg | ttgaggctgatgctgttgac |
| **Dkk1** | rat | cagctcaatcccaaggatgt | caggggagttccatcaagaa |
| **Dkk3** | rat | cctagacccagctgagttgc | gctactgggaaggagctgtg |
| **Txnip** | rat | ctgatggaggcacagtgaga | ctcgggtggagtgcttagag |
| **Cyba** | rat | ttgttgcaggagtgctcatc | cacggacagcagtaagtgga |
| **Dmnt1** | rat | ccaccaccaagctggtctat | tacggccaagttaggacacc |
| **Dnmt3a** | rat | cccaatggagacttggagaa | tcgatgttggtctgcttctg |
| **Dnmt3b** | rat | gtgaagcggatgatggagat | cctccgagacttggtagctg |
| **Tet1** | rat | tgtcacctgttgcatggatt | ttggatcttggctttcatcc |
| **Tet2** | rat | tcggaggagaagagtcagga | tagggcttgcattttccatc |
| **Tet3** | rat | acccggctctatgaaacctt | ggctgcagacttgatcttcc |
