# Supplementary material for: Tet3 mediates stable glucocorticoid-induced alterations in DNA methylation and Dnmt3a/Dkk1 expression in neural progenitors
Source: Cell Death Dis. 2015 Jun 18;6(6):e1793–. doi: 10.1038/cddis.2015.159 (PMC4669838; doi:10.1038/cddis.2015.159)
Supplement: Supplementary Table 2 [file cddis2015159x7.doc]

**Table S2** MSP primer sequences

| **Primer name** | forward | reverse |
| --- | --- | --- |
| **MDkk1** | ttggtagtcgagattttattcgtc | aaaaaaactacaaaaaaccatcgtt |
| **UDkk1** | ggtagttgagattttatttgttga | aaaaaaactacaaaaaaccatcatt |
| **MDkk3** | atagcgtttcgggatatataggc | tcctcctaaaaataattaaaaaccga |
| **UDkk3** | tgatagtgttttgggatatataggtg | ctcctaaaaataattaaaaaccaaa |
| **MTxnip** | tagttaatgggagaattgtgtacga | aaaaaaaatatcaaacgaaaaccg |
| **UTxnip** | tagttaatgggagaattgtgtatga | aaaaaaatatcaaacaaaaaccaaa |
| **MCyba** | tgcgtatttttaagtagggagtttc | ataaatacctcttccccaacgac |
| **UCyba** | ttttgtgtatttttaagtagggagtttt | aataaatacctcttccccaacaac |
