# Supplementary material for: Tet3 mediates stable glucocorticoid-induced alterations in DNA methylation and Dnmt3a/Dkk1 expression in neural progenitors
Source: Cell Death Dis. 2015 Jun 18;6(6):e1793–. doi: 10.1038/cddis.2015.159 (PMC4669838; doi:10.1038/cddis.2015.159)
Supplement: Supplementary Table 3 [file cddis2015159x8.doc]

| **Sample** | Con  1M NaCl | Con  Supernatant | Dex  1M NaCl | Dex  Supernatant |
| --- | --- | --- | --- | --- |
| **Total MBD-seq reads** | 20,632,719 | 34,476,024 | 11,289,181 | 21,825,305 |
| **Uniquely mapped reads** | 6,734,742 | 12,821,782 | 3,514,408 | 12,334,812 |
| **Distinct read position** | 6,290,132 | 12,198,958 | 3,361,152 | 11,704,649 |

**Table S3** Number of reads generated by MBD-Seq for each sample and sharing of peaks between samples.
